# Does continuous positive airway pressure therapy benefit patients with coronary artery disease and obstructive sleep apnea? A systematic review and meta‐analysis

**DOI:** 10.1002/clc.23669

**Published:** 2021-06-19

**Authors:** Yasha Chen, Yihong Chen, Feng Wen, Zhiqing He, Wenhao Niu, Changzhen Ren, Na Li, Qinqin Wang, Yusheng Ren, Chun Liang

**Affiliations:** ^1^ Department of Cardiology Shanghai Changzheng Hospital, Navy Medical University Shanghai China

**Keywords:** continuous positive airway pressure, coronary artery disease, meta‐analysis, obstructive sleep apnea

## Abstract

The prevalent co‐morbidity of coronary artery disease (CAD) and obstructive sleep apnea (OSA) has attracted great interest. However, effects of continuous positive airway pressure (CPAP) in patients with OSA and CAD for cardiovascular outcomes and deaths are still controversial. Usage of CPAP among patients with CAD and OSA could decrease the risk of cardiovascular events and death in adults. PubMed, EMBASE, Web of science, and Cochrane Library were systematically searched. Studies that described association of CPAP treatment with cardiovascular events in CAD and OSA patients were included. The main outcome was the major adverse cardiovascular events (MACE), including all‐cause death, cardiovascular death, myocardial infarction (MI), stroke, and repeat revascularization. Summary relative risks (risk ratios [RRs]) and 95% confidence intervals (CIs) of outcomes were pooled and heterogeneity was assessed with the I^2^ statistic. Nine studies enrolling 2590 participants with OSA and CAD were included and extracted data. There was significant association of CPAP with reduced risk of MACE (RR, 0.73, 95% CI [0.55, 0.96]), particularly among those with AHI less than 30 events/h (RR, 0.43, 95% CI [0.22, 0.84]). Similarly, the same result was found in all‐cause death (RR, 0.66, 95% CI, [0.46, 0.94]) and cardiovascular death (RR, 0.495, 95% CI [0.292, 0.838]). Our data suggested that CPAP usage, compared to usual care, was associated with reduced risks of cardiovascular outcomes or death in patients with OSA and CAD, particularly in the subgroup with AHI less than 30 events/h, which still needs further studies to confirm.

**Abbreviations**
ACS: acute coronary syndrome
AHI: apnea‐hypopnea index
BMI: body mass index
CAD: coronary artery disease
CI: confidence interval
CPAP: continuous positive airway pressure
ESSs: Epworth sleepiness scale score
HR: hazard ratio
MACE: major adverse cardiovascular events
MeSH: medical subject headings
MI: myocardial infarction
NOS: Newcastle‐Ottawa quality assessment scale
OSA: obstructive sleep apnea
PDD: portable diagnostic device
PG: polygraphy
PRISMA: systematic review and meta‐analysis
RCT: randomized controlled trail
RR: risk ratio

## 1 INTRODUCTION

Obstructive sleep apnea (OSA) is a common condition affecting 38%–65% of patients with coronary artery disease (CAD), an enormous health burden worldwide.^1^ Mechanisms linking OSA to CAD are not fully understood, but likely involve with sympathetic activation, vascular endothelial dysfunction, oxidative stress, systemic inflammation, coagulation, and metabolic dysregulation.^2^, ^3^ Current guidelines recommended continuous positive airway pressure (CPAP) therapy as standard treatment for patients with moderate to severe OSA.^4^, ^5^ However results of previous studies on the impact of CPAP on cardiovascular outcomes in OSA and CAD patients were conflicting. Some studies illustrated a beneficial effect,^6^, ^7^, ^8^, ^9^, ^10^, ^11^, ^12^ whereas two randomized controlled trials (RCTs) showed no significant effect on MACE.^13^, ^14^ The controversy stemming from the varying results causes confusion in everyday clinical practice about whether to use CPAP to provide cardiovascular protection or not. A few studies had conducted meta‐analyses trying to solve the above problem.^15^, ^16^, ^17^, ^18^ But the majority covered some cardiovascular diseases, which might cause heterogeneity and bias due to the diversity of research objects. And none included the ISAACC study,^14^ a multicenter randomized clinical trial, adding substantial new data in this area of research. In this systematic review, we conducted an updated meta‐analysis to determine the exact association of CPAP with risks of cardiovascular events and death in patients with OSA and CAD.

## 2 METHODS

### 2.1 Search strategy

We conducted the systematic review based on a predefined protocol and in accordance with preferred reporting items for systematic review and meta‐analysis (PRISMA) statement^19^ (eTable 1 in Supplement). Eligible studies were identified through a comprehensive literature search of electronic databases (PubMed, EMBASE, Web of Science, and the Cochrane Library) from inception through February, 2021, without any language restriction. Relevant text words and medical subject headings (MeSH) that consist of positive pressure respiration, sleep apnea, obstructive and coronary artery disease (see the eTable 2 in Supplement for detailed search strategy) were used. Moreover, potentially relevant reports identified from the reference lists of relevant studies, review articles, and chapters were hand‐searched and screened.

### 2.2 Inclusion and exclusion criteria

Studies considered for inclusion met the following criteria: (1) cohort studies including adults (age more than18 years) with OSA and CAD; (2) use of CPAP treatment compared with usual care; and (3) reported cardiovascular and mortality outcomes of interest. Exclusion criteria were as follows: (1) abstracts, letters, case reports, reviews or nonclinical studies; (2) studies with insufficient data for estimating hazard ratio (HR) and 95% confidence interval (CI); (4) studies had duplicate data or repeat analysis.

### 2.3 Data extraction and quality assessment

The review of potentially eligible scientific reports identified by the searches was completed by two authors (Yasha Chen and Yihong Chen) to identify reports for review in full text. Each full‐text article was then reviewed for eligibility and, for each included study, data recorded were (1) study characteristics: first author, year of publication, country, study phase and study design; (2) participants: sample size, age, sex, key inclusion criteria, apnea‐hypopnea index (AHI), Epworth Sleepiness Scale scores (ESSs); (4) interventions and comparison: CPAP equipment, type of interface, type of control, duration of treatment; and (5) outcomes: follow‐up duration, rate of lost‐to follow‐up and precision of measurements (risk ratios [RRs] or HRs with 95% CI) of each trial. Any disagreements would be resolved by discussion with a third party. Authors were contacted to clarify ambiguities and to request data on outcomes missing in primary reports.

To evaluate risk of bias, we used the individual criteria of the Newcastle‐Ottawa quality assessment scale (NOS)^20^ (eMethod in Supplement). There were three parts assessed: selection bias (0–4 points), comparability bias (0–2 points), and outcome assessment bias (0–3 points), which were each classified as low, unclear, or high. NOS scores of 6 were assigned as high‐quality studies.

### 2.4 Outcomes

The primary end point was the major adverse cardiovascular events (MACE), defined as a composite of all‐cause death, myocardial infarction (MI), stroke or repeat revascularization at the longest available follow‐up. The secondary end points were individual components of the primary end point. Definitions of events were in accordance to guidelines during each study period.

### 2.5 Statistical analysis

The numbers of dichotomous outcomes were summarized and mean values with standard deviations were collated for continuous outcomes. For every included trial, we retrieved or calculated the RRs and HRs with 95% CIs for the assessed outcomes. Fixed‐effects models assume that there is a common underlying effect and the variability observed is attributed to chance alone; random‐effects models acknowledge that true between‐study heterogeneity exists and take into account the presence of heterogeneity into their calculations. In the absence of heterogeneity, fixed‐ and random‐effects models yield the same results. We summarized RR or HR with a random‐effects model first and changed to a fixed‐effects model if no between study heterogeneity was found for the random‐effects model.^21^ And I^2^ statistic and was distinguished as low (I^2^ less than or equal to 25%), moderate (I^2^ less than 25% and more than 75%), or high (I^2^ equal to or more than 75%), as was a *p* value of less than or equal to .05 for heterogeneity.^22^, ^23^ Where there was a substantial heterogeneity (I^2^ more than 50%), subgroup analysis and sensitivity analyses was conducted for heterogeneity assessment. Subgroup analyses were conducted according to mean age, mean body mass index (BMI), mean AHI, OSA assessment, which might be potential mediators. In sensitivity analyses, we also excluded the following characteristics: (1) observational studies, (2) number of patients less than 200, (3) NOS score less than 9, (4) follow‐up duration no more than 36 months, and (5) lost to follow‐up rate more than 5%.

Funnel plots using the Egger test were visually assessed for publication bias.^24^ All tests were two‐tailed; *p* no more than .05 was considered statistically significant. Statistical analyses were performed with Stata/SE version 15.1.

## 3 RESULTS

### 3.1 Study selection and baseline characteristics

**FIGURE 1:**
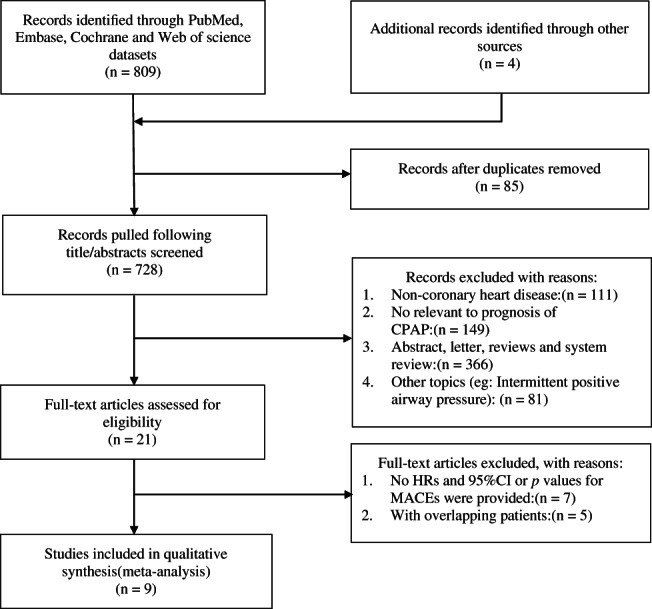
Study identification and selection. Flow chart for the systematic review and meta‐analysis as per the preferred reporting. System for systematic review and meta‐analysis (PRISMA)

Among the retrieved 813 articles, 85 were excluded because of duplication, and 728 articles were excluded after review of the title or abstract, 21 articles were reviewed in full text, and nine studies (2590 participants) met the inclusion criteria. (Figure 1).

The summary and baseline characteristics of patients included in these trials are shown in Table 1. The meta‐analysis included two retrospective cohort studies,^7^, ^11^ four prospective cohort studies^6^, ^8^, ^9^, ^12^ and three RCTs^10^, ^13^, ^14^ published from 2004 to 2020. All studies except one^14^ were single‐center trials. The median of sample size was 129 (range from 46 to 1255). The median of average age of study participants was 60.3 years (range of average age from 55.1 to 68.3 years) and 83.3% of participants were men (range from 82.2% to 98.1%). The median of average BMI of patients was 28.3 (range from 27.3 to 34.1) and the median of AHI was 30.6 events/h (range from 21.7 to 42.8 events/h). All participants enrolled in these studies were diagnosed with OSA and CAD. Diagnosis of OSA was based on polysomnography in all but and two trials^9^, ^11^ that used portable diagnostic device (PDD), with AHI equal to or more than 15 as cutoff value in most studies. Participants received standard intervention of CPAP in most studies. Usual care served as a control in all trials. The median duration of follow‐up was 57.6 months from 36 months to 86.5 months, and a small proportion of patients were lost to follow‐up (the maximum utmost up to 10%). The primary end point (MACE) was reported in all studies except for one,^8^ which only reported MI and repeat revascularization.

**TABLE 1:** Main characteristics of the eligible clinical trials

Source	Study design, study period	No. of participants (CPAP/control)	Mean age (years)	Male (%)	Mean BMI (kg/m^2^)	Mean AHI (events/h)	Mean ESS (points)	OSA assessment	Key inclusion criteria	CPAP duration (h/d)	Follow‐up duration (months)	Rate of loss to follow‐up (%)
Milleron et al. 2004 (Frace)	Prospective cohort, single‐center, 1991–1999	54 (25/29)	57.3	98.1	28.3	31.2	NR	PG	CAD, OSA (AHI ≥ 15/h)	≥3.0	86.5	5.6
Cassar et al. 2007 (American)	Retrospective cohort, single‐center, 1992–2004	371 (175/196)	64.0	87.6	34.1	44.2	NR	PG	CAD, OSA (AHI ≥ 15/h)	NR	60.0	10.0
Garcia‐Rio et al. 2013 (Spain)	Prospective cohort, single‐center, 2003–2005	123 (71/52)	58.0	86.2	27.3	21.7	8.5	PG	MI, OSA (AHI ≥ 5/h)	≥3.5	78.0	2.4
Capodanno et al. 2014 (Italy)	Prospective cohort, single‐center, 2008	129 (17/112)	68.3	80.6	27.3	22.4	7.0	PDD	CAD, OSA (AHI ≥ 15/h)	NR	36.0	0.0
Huang et al. 2015 (China)	RCT, single‐center, 2009–2012	73 (37/36)	62.3	82.2	27.7	28.5	8.8	PG	CAD, hypertension, OSA (AHI ≥ 15/h)	≥4.0	36.0	2.4
Wu et al. 2015 (China)	Retrospective cohort, single‐center, 2002–2012	295 (128/167)	55.1	84.4	29.7	42.8	NR	PG or PDD	CAD, OSA (AHI ≥ 15/h)	≥4.0	57.6	1.5
Leão et al. 2016 (Portugal)	Prospective cohort, single‐center, NR	46 (19/27)	63.5	82.6	27.8	30.6	8.8	PDD	ACS, OSA (AHI ≥ 5)	≥4.0	75.0	0.0
RICCADSA. 2016 (Sweden)	RCT, single‐center, 2005–2010	244 (122/122)	66.0	84.1	28.5	28.8	5.5	PG	CAD, OSA (AHI ≥ 15/h, EES < 10)	≥4.0	56.9	0.4
ISAACC. 2020 (Spain)	RCT, multicenter, 2011–2018	1255 (629/626)	60.3	84.3	29.5	40.0	5.3	PG	ACS, OSA (AHI ≥ 15)	≥4.0	40.2	2.1

Abbreviations: ACS, acute coronary syndromes; AHI, apnea‐hypopnea index; BMI, body mass index; CAD, coronary artery disease; CPAP, continuous positive airway pressure; ESS, Epworth sleepiness scale score; OSA, obstructive sleep apnea; PDD, portable diagnostic device; PG, polygraphy; RCT, randomized controlled.

### 3.2 Association of CPAP with cardiovascular outcomes and deaths

#### 3.2.1 Primary endpoint

**FIGURE 2:**
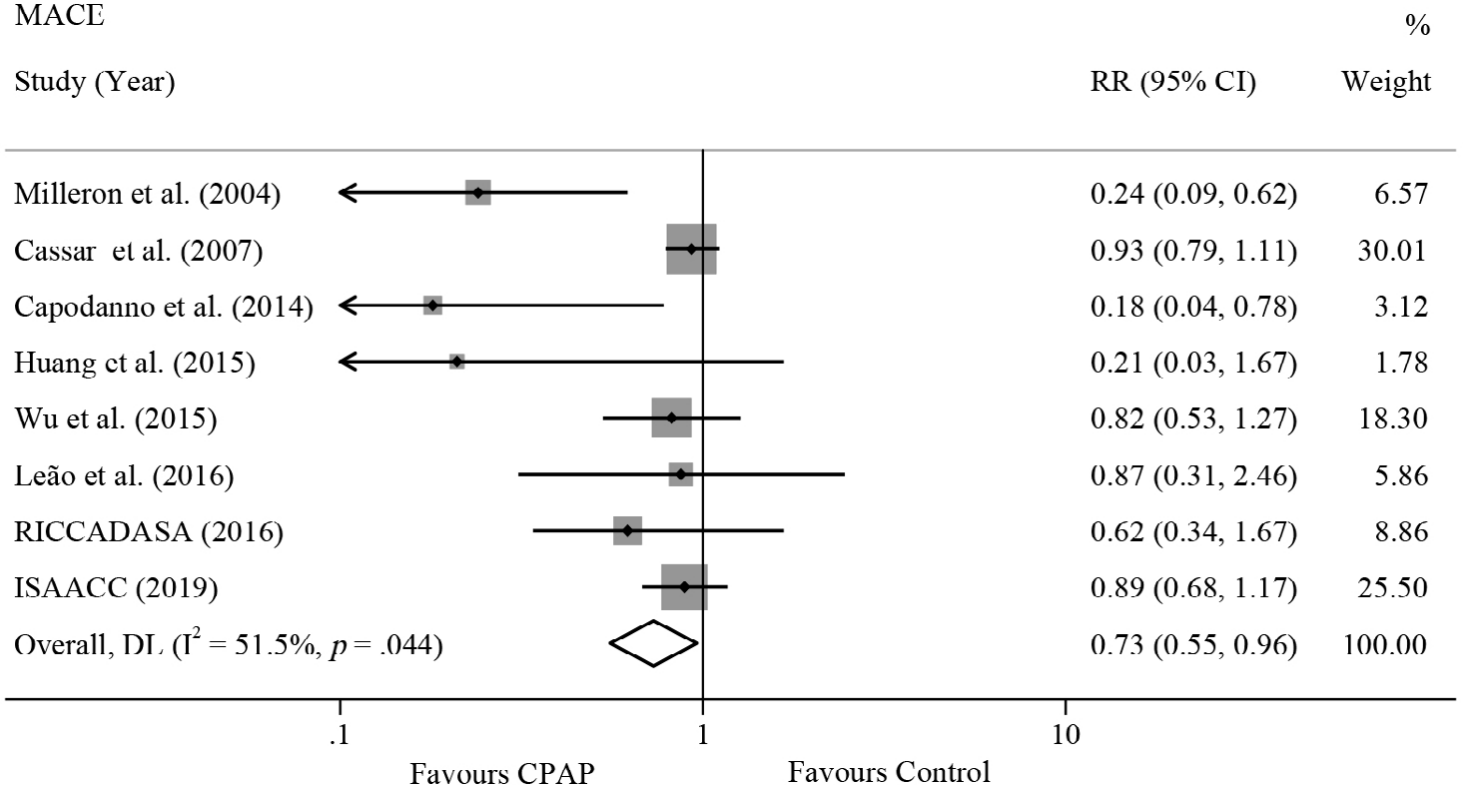
Forest plot for the association between CPAP and risk of MACE. The area of the square represents the point of estimate is proportional to the weight of study. Each point on the figure represents a RR. The diamond represents the pooled estimate of effect, as calculated according to the random effects model. RR < 1 means a lower risk with CPAP, and RR > 1means a higher risk with CPAP. 95% CI does not include the number 1 means statistical difference between the two groups. CI, confidence interval; CPAP, continuous positive airway pressure; MACE, major adverse cardiovascular event; RR, risk ratio

Pooling data from eight studies (five observational studies and three RCTs)^6^, ^7^, ^9^, ^10^, ^11^, ^12^, ^13^, ^14^ with 2467 participants demonstrated significant associations of CPAP with MACE (RR, 0.73,95% CI, [0.55, 0.96]) by random‐effects models with a moderate degree of heterogeneity (I^2^ = 51.5%, *p* = .04) (Figure 2). This result was driven by the study of Cassar et al.,^7^ which carried 30.01% of the weight.

Particularly, a more significant difference was found in AHI less than 30 events/h subgroup (RR, 0.43, 95% CI [0.22, 0.84]). Heterogeneity reduction was showed in the analysis of primary endpoint for both mean AHI equal to or more than 30 events/h subgroup and mean AHI less than 30 events/h subgroup. But satisfactory source of heterogeneity and significant difference were not found out by the following subgroup analyses: mean age (more than or equal to 60 years or less than 60 years), mean BMI (more than or equal to 28 kg/m^2^ or less than 28 kg/m^2^), OSA assessment (PSG or PDD) (eTable 3 and eFigure 1 in Supplement).

In sensitivity analyses of MACE, there was no substantive difference while excluding the following characteristics: (1) observational studies (RR, 0.83, 95% CI, [0.65, 1.08]), (2) number of patients less than 200 (RR, 0.90, 95% CI, [0.78, 1.03]), (3) NOS score less than 9 (RR, 0.57, 95% CI, [0.28, 1.17]), (4) follow‐up duration shorter than 36 months (RR, 0.87, 95% CI, [0.76, 1.00]), and (5) lost to follow‐up rate more than 5% (RR, 0.81, 95% CI, [0.65, 1.00]). And the heterogeneity was attenuated in most of those groups (eTable 4 and eFigure 2 in Supplement).

#### 3.2.2 Secondary endpoints

**FIGURE 3:**
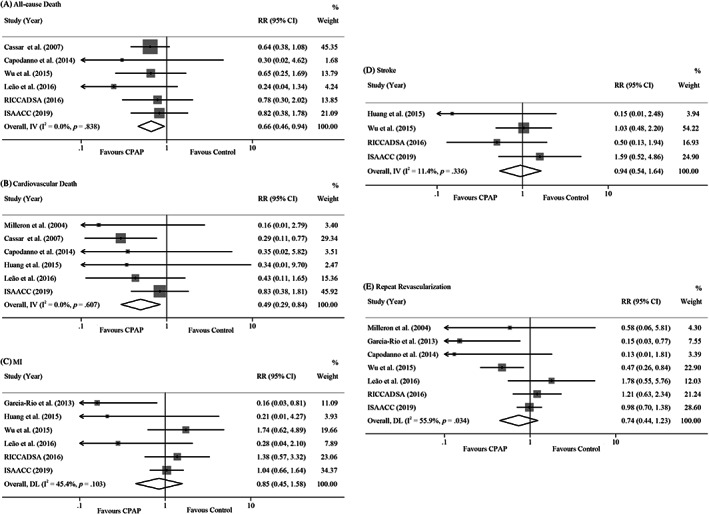
Forest plots for the association between CPAP and risk of (A) all‐cause death, (B) cardiovascular death, (C) myocardial Infarction (MI), (D) stroke, or (E) repeat revascularization. Each point on the figure represents a RR. The diamond represents the pooled estimate of effect, as calculated according to the random effects model first and changed to a fixed‐effects model if no significant heterogeneity was found for the random‐effects model. RR < 1 means a lower risk with CPAP, and RR > 1means a higher risk with CPAP. 95% CI does not include the number 1 means statistical difference between the two groups. CI, confidence interval; CPAP, continuous positive airway pressure; MI, myocardial infarction; RR, risk ratio

Analyses of all‐cause death, cardiovascular death, MI, stroke and repeat revascularization were shown in Figure 3. With respect to all‐cause death, data were available from six studies^7^, ^9^, ^11^, ^12^, ^13^, ^14^ with 2340 participants (Figure 3(A)). When pooled, CPAP treatment was associated with a significantly lower risk of all‐cause death (RR, 0.66, 95% CI, [0.46, 0.94], I^2^ = 0, *p* = .009). Cardiovascular death was evaluated in six trials^6^, ^7^, ^9^, ^10^, ^12^, ^14^ with a total of 1928 participants. When combined, CPAP was significantly associated with a reduced risk for cardiovascular death (RR, 0.495, 95% CI, [0.292, 0.838], I^2^ = 0, *p* = .838). (Figure 3(B)). Six studies^8^, ^10^, ^11^, ^12^, ^13^, ^14^ (2036 patients) reported on MI. However, there was no significant difference in the risk of MI with CPAP (RR, 0.845, 95% CI, [0.451, 1.582], I^2^ = 45.4%, *p* = .103) (Figure 3(C)). For stroke, when four studies^10^, ^11^, ^13^, ^14^ were pooled (1867 participants), the risk of stroke was no reduced with CPAP treatment (RR, 0.941, 95% CI [0.539, 1.643], I^2^ = 11.40%, *p* = .336) (Figure 3(D)). For repeat revascularization, when seven studies^6^, ^8^, ^9^, ^11^, ^12^, ^13^, ^14^ (2146 patients) were pooled, CPAP was not associated with the risk of repeat revascularization (RR, 0.73, 95% CI, [0.442, 1.231]), and the pooled estimate showed substantial heterogeneity (I^2^ = 55.9%, *p* = .034) (Figure 3(E)).

Subgroup analysis showed that the risk of repeat revascularization significant decreased in mean age less than 60 years subgroup (RR, 0.420, 95% CI, [0.246, 0.719]), but did not decrease in mean age equal to or more than 60 years subgroup (RR, 1.033, 95% CI, [0.773, 1.38]). And the heterogeneity was attenuated in both subgroups (0% and 14.7%). Other subgroup analysis did not find out the satisfactory source for this heterogeneity. (eTable 5 and eFigure 3 in Supplement). Sensitivity analysis was performed to assess the heterogeneity of risk of repeat revascularization. While excluding the studies for observational study, number of participants less than 200 and follow‐up duration less than or equal to 36 months respectively, the heterogeneity was attenuated (eTable 6 and eFigure 4 in Supplement).

### 3.3 Qualitative assessment and bias assessment

**FIGURE 4:**
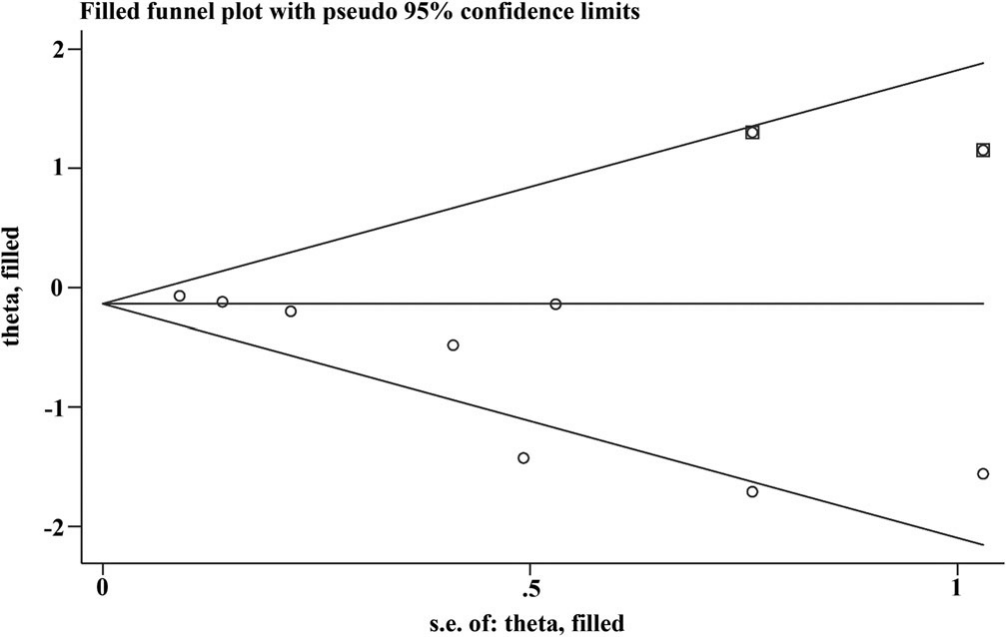
Filled funnel plot of RRs from studies for the association between CPAP and the risk of MACE. The circles alone are real studies and the circles enclosed in boxes are “filled” studies. The horizontal line represents the summary effect estimates, and the diagonal lines represent pseudo‐95% CI limits. CI, confidence interval; CPAP, continuous positive airway pressure; MACE, major adverse cardiovascular event; RR, risk ratio

All studies showed high quality (NOS score equal to or more than 6) (eFigure 5 in Supplement).While the funnel plot (eFigure 6 in Supplement) suggested possible publication bias and Egger's test was statistically significant for publication bias (*p* = .001) (eFigure 7 in Supplement) for primary end point, the nonparametric trim and fill estimate^25^ of the effects of publication bias (Figure 4), produced essentially the same results as the meta‐analysis of the published studies: RR 0.731 (95% CI, [0.555, 0.963]) for actual studies versus RR 0.730 (95% CI, [0.553, 0.963]) from the fill and trim analysis, suggesting that publication bias is not likely to explain our findings.

## 4 DISCUSSION

A large number of studies have investigated the effects of CPAP in cardiovascular outcomes. Therefore, we reviewed the published studies and undertook an updated meta‐analysis to make a more precise estimation of the benefit of CPAP for cardiovascular outcomes in patients with OSA and CAD.

In this meta‐analysis of nine studies involving 2590 participants, we have demonstrated a decreased risk of MACE associated with CPAP, especially in AHI less than 30 events/h subgroup. Additionally, the results were nearly the same in individual outcomes including all‐cause death and cardiovascular death. Indeed, CPAP is associated with at least 37% decrease in the risk of MACE compared to control patients in usual care group. Yet our analysis demonstrated null result with respect to MI, stroke or repeat revascularization.

The negative results of included studies were mainly derived from RICCADSA study^13^ and ISAACC study.^14^ RICCADSA trial, a single‐center RCT from Sweden, illustrated a viewpoint that routine CPAP did not significantly reduce the adverse cardiovascular outcomes in patients with non‐sleepy OSA (HR 0.80 (95% CI, [0.46, 1.41], *p* = .449). However, A significant beneficial effect of CPAP was seen in patients used the device for equal to or more than 4 h/night, with an adjusted HR that similar to a prior observational study.^26^ Another RCT of 1255 patients with ACS at 15 hospitals across Spain, ISAACC study, showed that CPAP treatment did not result in a significantly lower prevalence of cardiovascular events in patients with moderate to severe OSA. But the patients in this study did not attend any sleep unit, but were instead recruited from coronary units, without being referred for a sleep‐related breathing problem or symptoms. In such population, getting a good adherence to CPAP treatment was very difficult. Hence, this population had a very low compliance to CPAP treatment (2.78 h/night), the benefit effect of sustained well‐conducted CPAP (more than or equal to 4 h/night) could not be excluded. A greater effect on patients with adequate adherence than on those without adequate adherence has been illuminated.^27^, ^28^, ^29^ The SAVE trial study, a large‐scale RCT of 2602 CAD patients reported better outcomes among patients who were adherent to CPAP therapy (equal to or more than 4 hours per night) than those who did not receive CPAP or who used CPAP less than 4 hours per night. A prior meta‐analysis of RCTs for CAD patients showed that utilization of CPAP in patients with OSA was not associated with reduced risk of major adverse cardiac events, but wearing CPAP more than 4 hours did in fact decrease MACE in the subgroup analysis.^16^ This might be derived from low average CPAP usage achieved in most trials. We speculated that poor CPAP treatment adherence might be the major reason for above null outcomes. Adherence of CPAP still remains a challenging in this field. However, the null result of two RCTs, the final combined primary endpoint still showed a positive effect for CPAP. The reason behind it might be the significant benefit observed in observational studies. Considering the poor compliance and limitation of observative study, further RCTs on patients with good CPAP compliance are required.

Underlying mechanisms between OSA and cardiovascular outcomes are still elusive. Apnea and hypopneas were suggested to trigger endothelial function impairment, oxidative stress, systemic inflammation, and pressure change in thorax and cardiac chambers,^30^, ^31^, ^32^ which might induce functional change and structural damage of the coronary arteries, accelerate atherosclerotic process, and finally lead to CAD. OSA might over‐activate the sympathetic nervous system and promote the release of vasoconstrictive substances such as catecholamines, angiotensin II, endothelin, and so on.^33^, ^34^, ^35^ These pathophysiological changes could increase night blood pressure, heart rate and result in an increase in myocardial oxygen demand, cardiac load, and eventually lead to cardiovascular events.^36^, ^37^ CPAP has been demonstrated to reduce circulating inflammatory and thrombogenic factors, decrease oxidative stress and improve endothelial dysfunction, and down‐regulate sympathetic nerve activity.^38^ Due to the ability to reverse these dysfunctions, CPAP has the potential to impede the progression of cardiovascular disease such as atherosclerosis.^39^

More interestingly, our review showed lower AHI had a significant association with reduced MACE (RR, 0.43, 95% CI [0.22, 0.84]). Central obesity, which generates fat distribution particularly at the abdominal level, upper body and neck, is the most significant predisposing factor for OSA.^40^, ^41^ Further, increased BMI were independent and significantly correlated to a greater AHI.^42^ Result of a recent randomized study illustrated that positive airway pressure might lead to volume retention among obese participants with OSA, exerting adverse health effects in this vulnerable population.^43^ This supported our result to some extent. But the mean BMI of most included studies in our review did not meet the criteria of obesity. Hence, it is not sufficient to justify the negative outcome in obese population. Given that, further studies investigating CPAP's effect on obesity patients are in great need.

Though prior meta‐analysis existed, and the final result was roughly consistent with it.^15^ But our meta‐analysis has several substantial strengths. First, compared to prior meta‐analysis, which is mainly compose of observational studies, we included a multicenter RCT——ISAACC study,^14^ providing more precise and substantial data for further evaluation. Second, a Japanese observational study was excluded for poor quality and not consistent with the aim of our meta‐analysis, which only evaluated OSA's effects on patients with IM.^44^ Third, while prior meta‐analysis retrieved by Myocardial ischemia, while previous reviews retrieved literature based on the keyword “Myocardial ischemia,” our retrieval strategy was changed to “Coronary artery disease,” which helped us to obtain target references more accurately. Fourthly, we conducted comprehensive subgroup and sensitivity analysis that explained the potential heterogeneity and enhanced the precision of merged results. In AHI less than 30 events/h subgroup, we found striking benefits of CPAP treatment. That might provide new therapeutic strategies and inspire new researches for those vulnerable patients.

Despite the robustness of data from our study, it still has several limitations. The most important one is the moderate‐to‐high heterogeneity derived from the included trials. As the characteristics of enrolled participants, study designs, sample sizes, follow‐up durations, loss to follow‐up rates were varied, it was not surprising that all included studies fell into remarkable heterogeneity. Reassuringly, sensitivity analysis showed that results were not significantly altered and heterogeneity was reduced by excluding studies at high risk of bias. Second, studies included in this meta‐analysis were inconsistent in OSA measurement, as six studies measured OSA with polygraphy (PG), three measured OSA in with PDD. The results of the study might be influenced by the different measuring method. Third, all included trials compared therapeutic CPAP to usual care rather than placebo CPAP, which might increase the risk of attrition bias and detection bias. Finally, the eligible studies were relatively small in the establishment of prognostic values. Egger's test has relatively lower power when the number of studies included in meta‐analysis is less than 10. Though the fill and trim analysis suggested that publication bias might be not likely to explain our findings, the result of publication bias might not be accurate.

## 5 CONCLUSION

The use of CPAP, compared to usual care, was associated with reduced risks of cardiovascular outcomes or death in patients with OSA and CAD, particularly in the subgroup with AHI less than 30 events/h. Further studies are needed to evaluate the benefits of CPAP for prevention of cardiovascular outcomes.

## CONFLICT OF INTEREST

All authors declared that they have no conflict of interest.

## AUTHOR CONTRIBUTIONS

Yihong Chen contributed to study concept and manuscript preparation. Yasha Chen and Feng Wen contributed to data collection, data analysis, and drafting the manuscript. Wenhao Niu, Changzhen Ren, Na Li, Qinqin Wang, Yusheng Ren, Chun Liang, and Zhiqing He contributed to revise the manuscript critically for important intellectual content. All authors had given final approval of the version to be published.

## Supporting information

**Appendix** S1: Supporting Information

## Data Availability

All data generated or analysed during this study are included in this published article (and its supplementary information files).
