# Supplementary material for: Does continuous positive airway pressure therapy benefit patients with coronary artery disease and obstructive sleep apnea? A systematic review and meta‐analysis
Source: Clin Cardiol. 2021 Jun 19;44(8):1041–9. doi: 10.1002/clc.23669 (PMC8364731; doi:10.1002/clc.23669)
Supplement: Supplementary file 1 — Appendix S1: Supporting Information [file CLC-44-1041-s001.pdf]

Supplementary Materials

eMethod. Study Quality Assessment

eTable 1. PRISMA Checklist

eTable 2. Literature Search

eTable 3. Subgroups Analyses for the Association Between CPAP and Risk of MACE

eTable 4. Sensitivity Analyses for the Association Between CPAP and Risk of MACE

eTable 5. Subgroups Analyses for the Association Between CPAP and Risk of Repeat Revascularization

eTable 6. Sensitivity Analyses for the Association Between CPAP and Risk of Repeat Revascularization

eFigure 1. Forest Plots of the Association Between CPAP and Risk of MACE for Subgroup Analyses

eFigure 2. Forest Plots of the Association Between CPAP and Risk of MACE for Sensitivity Analyses

eFigure 3. Forest Plots of the Association Between CPAP and Risk of Repeat Revascularization for Subgroup Analyses

eFigure 4. Forest Plots of the Association Between CPAP and Risk of Repeat Revascularization for Sensitivity Analyses

eFigure 5. Qualitative assessment of trials and exploration of potential sources of bias.

eFigure 6. Funnel Plot for Publication bias on the Association between CPAP and Risk of MACE.

eFigure 7. Egger's Funnel Plot for Publication bias on the Association between CPAP and Risk of MACE.

eMethods: Study Quality Assessment

The Newcastle-Ottawa Quality Assessment Scale items assessed were: 1-2) representativeness of the exposed/unexposed cohort (population-based vs. convenience-based), 3) ascertainment of exposure (thyroid function measurement), 4) availability of relevant confounders for adjustment, 5) assessment of outcome with objective fracture assessment (defined as using radiographic images and/or reviewing radiologists or orthopedic reports and other support documents to verify clinical diagnoses; or record linkage on database records) and formal adjudication procedure (defined as using predefined criteria applied by experts), 6) length of follow-up period, and 7) loss to follow-up. We did not list the presence of outcome (current fracture) at cohort study start, as we excluded participants with acute fracture at baseline from the analysis. Study quality was assessed independently by two authors (M.R.B and T.H.C.).

eTable 1. PRISMA Checklist.

Section/topic	#	Checklist item	Reported on page #
**TITLE**			
Title	1	Identify the report as a systematic review, meta-analysis, or both.	1
**ABSTRACT**			
Structured summary	2	Provide a structured summary including, as applicable: background; objectives; data sources; study eligibility criteria, participants, and interventions; study appraisal and synthesis methods; results; limitations; conclusions and implications of key findings; systematic review registration number.	3
**INTRODUCTION**			
Rationale	3	Describe the rationale for the review in the context of what is already known.	5
Objectives	4	Provide an explicit statement of questions being addressed with reference to participants, interventions, comparisons, outcomes, and study design (PICOS).	6
**METHODS**			
Protocol and registration	5	Indicate if a review protocol exists, if and where it can be accessed (e.g., Web address), and, if available, provide registration information including registration number.	6
Eligibility criteria	6	Specify study characteristics (e.g., PICOS, length of follow-up) and report characteristics (e.g., years considered, language, publication status) used as criteria for eligibility, giving rationale.	6
Information sources	7	Describe all information sources (e.g., databases with dates of coverage, contact with study authors to identify additional studies) in the search and date last searched.	7
Search	8	Present full electronic search strategy for at least one database, including any limits used, such that it could be repeated.	6
Study selection	9	State the process for selecting studies (i.e., screening, eligibility, included in systematic review, and, if applicable, included in the meta-analysis).	6
Data collection process	10	Describe method of data extraction from reports (e.g., piloted forms, independently, in duplicate) and any processes for obtaining and confirming data from investigators.	7
Data items	11	List and define all variables for which data were sought (e.g., PICOS, funding sources) and any assumptions and simplifications made.	6
Risk of bias in individual studies	12	Describe methods used for assessing risk of bias of individual studies (including specification of whether this was done at the study or outcome level), and how this information is to be used in any data synthesis.	7
Summary measures	13	State the principal summary measures (e.g., risk ratio, difference in means).	7, 8
Synthesis of results	14	Describe the methods of handling data and combining results of studies, if done, including measures of consistency (e.g., I^2) for each meta-analysis.	8

Section/topic	#	Checklist item	Reported on page #
Risk of bias across studies	15	Specify any assessment of risk of bias that may affect the cumulative evidence (e.g., publication bias, selective reporting within studies).	9
Additional analyses	16	Describe methods of additional analyses (e.g., sensitivity or subgroup analyses, meta-regression), if done, indicating which were pre-specified.	8, 9
**RESULTS**			
Study selection	17	Give numbers of studies screened, assessed for eligibility, and included in the review, with reasons for exclusions at each stage, ideally with a flow diagram.	9, 10
Study characteristics	18	For each study, present characteristics for which data were extracted (e.g., study size, PICOS, follow-up period) and provide the citations.	9,10,26
Risk of bias within studies	19	Present data on risk of bias of each study and, if available, any outcome level assessment (see item 12).	10-12
Results of individual studies	20	For all outcomes considered (benefits or harms), present, for each study: (a) simple summary data for each intervention group (b) effect estimates and confidence intervals, ideally with a forest plot.	10-12
Synthesis of results	21	Present results of each meta-analysis done, including confidence intervals and measures of consistency.	10-12
Risk of bias across studies	22	Present results of any assessment of risk of bias across studies (see Item 15).	13
Additional analysis	23	Give results of additional analyses, if done (e.g., sensitivity or subgroup analyses, meta-regression [see Item 16]).	10-12
**DISCUSSION**			
Summary of evidence	24	Summarize the main findings including the strength of evidence for each main outcome; consider their relevance to key groups (e.g., healthcare providers, users, and policy makers).	13-15
Limitations	25	Discuss limitations at study and outcome level (e.g., risk of bias), and at review-level (e.g., incomplete retrieval of identified research, reporting bias).	16
Conclusions	26	Provide a general interpretation of the results in the context of other evidence, and implications for future research.	17
**FUNDING**			
Funding	27	Describe sources of funding for the systematic review and other support (e.g., supply of data); role of funders for the systematic review.	18

eTable 2. Literature Research (take PubMed for example).

	Key words	Results(n)
**#1**	Positive Pressure Respiration [MeSH Major Topic]	16,072
**#2**	Sleep Apnea, Obstructive [MeSH Major Topic]	19,098
**#3**	coronary artery disease [MeSH Major Topic]	53,879
**#4**	((((((((((((((Positive Pressure Respiration) OR (Positive-Pressure Respirations)) OR (Respiration, Positive-Pressure)) OR (Respirations, Positive-Pressure)) OR (Positive-Pressure Ventilation)) OR (Positive Pressure Ventilation)) OR (Positive-Pressure Ventilations)) OR (Ventilation, Positive-Pressure)) OR (Ventilations, Positive-Pressure)) OR (Positive End-Expiratory Pressure)) OR (End-Expiratory Pressure, Positive)) OR (End-Expiratory Pressures, Positive)) OR (Positive End Expiratory Pressure)) OR (Positive End-Expiratory Pressures)) OR (Pressure, Positive End-Expiratory)) OR (Pressures, Positive End-Expiratory)	34869
**#5**	(((((((((((Apneas, Obstructive Sleep) OR (Obstructive Sleep Apneas)) OR (Sleep Apneas, Obstructive)) OR (Obstructive Sleep Apnea Syndrome)) OR (Obstructive Sleep Apnea)) OR (OSAHS)) OR (Syndrome, Sleep Apnea, Obstructive)) OR (Sleep Apnea Syndrome, Obstructive)) OR (Apnea, Obstructive Sleep)) OR (Sleep Apnea Hypopnea Syndrome)) OR (Syndrome, Obstructive Sleep Apnea)) OR (Upper Airway Resistance Sleep Apnea Syndrome)) OR (Syndrome, Upper Airway Resistance, Sleep Apnea)	509
**#6**	(((((((((((Artery Disease, Coronary) OR (Artery Diseases, Coronary)) OR (Coronary Artery Diseases)) OR (Disease, Coronary Artery)) OR (Diseases, Coronary Artery)) OR (Coronary Arteriosclerosis)) OR (Arterioscleroses, Coronary)) OR (Coronary Arterioscleroses)) OR (Atherosclerosis, Coronary)) OR (Atheroscleroses, Coronary)) OR (Coronary Atheroscleroses)) OR (Coronary Atherosclerosis)) OR (Arteriosclerosis, Coronary)	201915
**#7**	#1 OR #4	34869
**#8**	#2 OR #5	509
**#9**	#3 OR #6	201915
**#10**	#7 AND #8 AND #9	130

eTable 3. Subgroups Analyses for the Association Between CPAP and Risk of MACE

Subgroup	No. of Studies	Reference	RR (95%CI)	Heterogeneity		P_{eff}
				I^2	P_{het}	
**Mean age（years）**						
Mean age < 60	2	9,11	0.480(0.145,1.584)	80.70%	0.023	0.228
Mean age ≥ 60	6	7, 9, 10, 12, 13, 14	0.826(0.644,1.058)	33.20%	0.187	0.131
**Mean BMI (kg/m2)**						
Mean BMI ≥ 28	5	6, 7, 11, 13, 14	0.802(0.625,1.029)	51.30%	0.084	0.082
Mean BMI < 28	3	9, 10, 12	0.382(0.125,1.173)	44.00%	0.168	0.093
**Mean AHI (events/h)**						
Mean AHI ≥ 30	5	6, 7, 11, 12, 14	0.883(0.769,1.013)	46.60%	0.112	0.076
Mean AHI < 30	3	9, 10, 13	0.430(0.220,0.837)	22.20%	0.277	0.013
**OSA Assessment**						
PSG	5	6, 7, 10, 13, 14	0.731(0.520,1.029)	60.20%	0.040	0.072
PDD	3	9, 11, 12	0.642(0.314,1.313)	46.50%	0.154	0.224

eTable 4. Sensitivity Analyses for the Association Between CPAP and Risk of MACE

Subgroup	No. of Studies	Reference	RR (95%CI)	Heterogeneity		P_{eff}
				I^2	P_{het}	
excluding No. of Participants < 200	4	7, 11, 13, 14	0.897(0.782,1.030)	0.00%	0.757	0.125
excluding observational study	3	10, 13, 14	0.835(0.645,1.080)	20.40%	0.284	0.170
excluding NOS score＜9	3	6, 13, 14	0.571(0.279,1.165)	71.00%	0.032	0.124
excluding follow-up duration ≤ 36 months	6	6, 7, 11, 12, 13, 14	0.874(0.763,1.001)	39.10%	0.145	0.051
excluding lost to follow-up rate＞5%	6	9, 10, 11, 12, 13, 14	0.806(0.651,0.999)	22.80%	0.262	0.049

eTable 5. Subgroups Analyses for the Association Between CPAP and Risk of Repeat Revascularization

Subgroup	No. of Studies	Reference	RR (95%CI)	Heterogeneity		
				I^2	P_{het}	P_{eff}
**Mean age (years)**						
Mean age < 60	3	6, 8, 11	0.420(0.246,0.719)	0.00%	0.419	0.002
Mean age≥60	4	9, 12, 13, 14	1.033(0.773,1.381)	14.70%	0.318	0.825
**Mean BMI (kg/m2)**						
Mean BMI ≥ 28	4	6, 11, 13, 14	0.822(0.525,1.288)	48.20%	0.122	0.393
Mean BMI < 28	3	8, 9, 13, 14	0.390(0.058,2.633)	72.80%	0.025	0.334
**Mean AHI (events/h)**						
Mean AHI ≥ 30	4	6, 11, 12, 14	0.817(0.470,1.421)	51.80%	0.101	0.474
Mean AHI < 30	3	8, 9, 13	0.368(0.067,2.021)	72.80%	0.025	0.250
**OSA Assessment**						
PSG	4	6, 8, 13, 14	0.838(0.466,1.504)	46.60%	0.132	0.553
PDD	3	9, 11, 12	0.630(0.199,1.994)	61.80%	0.073	0.432

eTable 6. Sensitivity Analyses for the Association Between CPAP and Risk of Repeat Revascularization

Subgroup	No. of Studies	Reference	RR (95%CI)	Heterogeneity		
				I^2	P_{het}	P_{eff}
excluding observational study	2	13, 14	1.024(0.758,1.385)	0.00%	0.577	0.876
excluding No. of Participants＜200	3	11, 13, 14	0.830(0.501,1.375)	64.80%	0.059	0.470
excluding NOS score＜9	4	6, 9, 13, 14	0.838(0.466,1.504)	46.60%	0.132	0.553
excluding follow-up duration ≤ 36 months	5	6, 8, 12, 13, 14	0.947(0.569,1.576)	39.80%	0.156	0.835
excluding lost to follow-up rate＞5%	6	9, 10, 11, 12, 13, 14	0.738(0.427,1.277)	63.00%	0.019	0.277

eTable3-6, RR, relative risk; CI, confidential interval; P_{het}, P value of heterogeneity test; P_{eff}, P value of pooled effect, P value of pooled effect; NOS, Newcastle-Ottawa Scale.

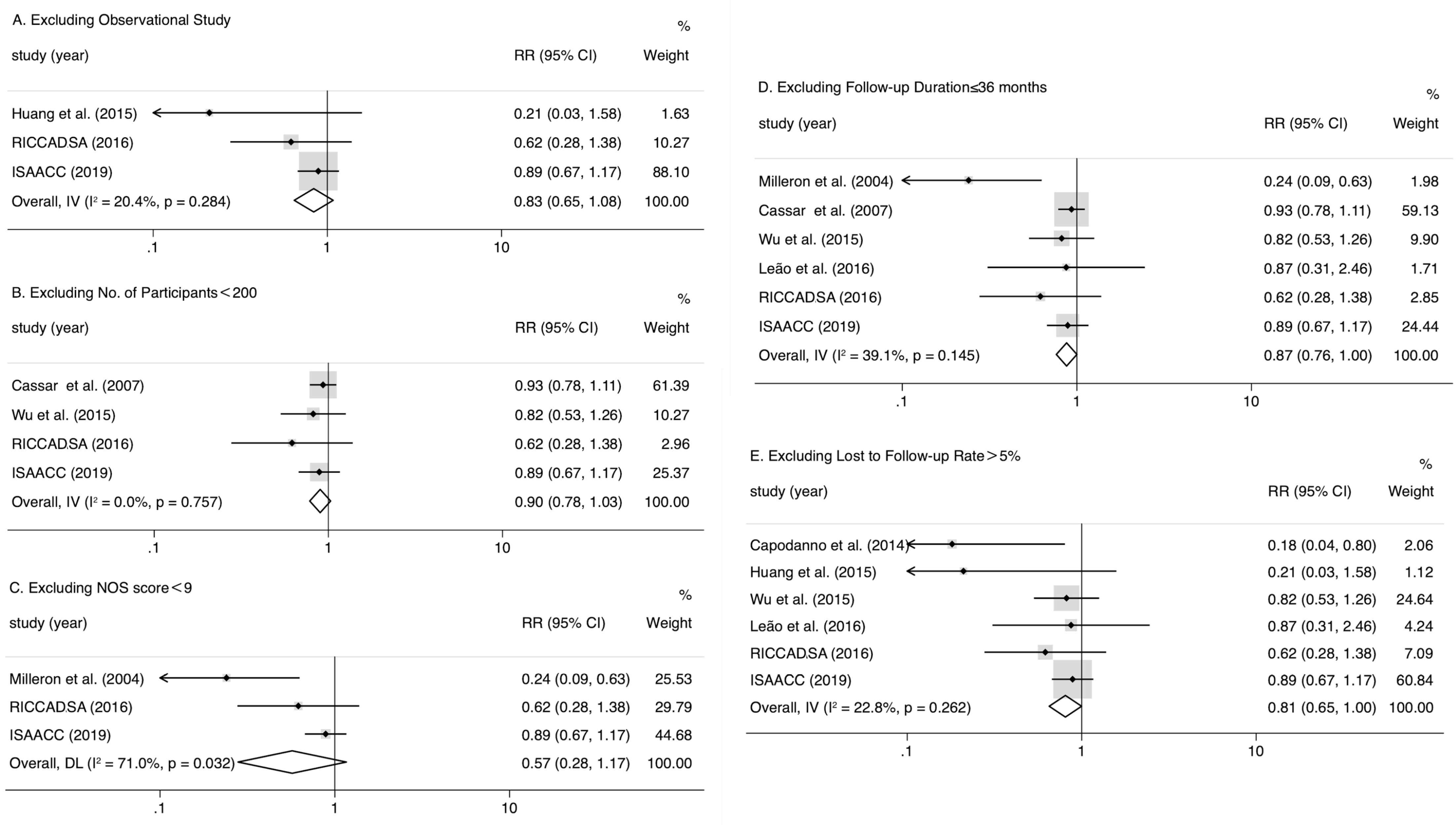

A. Excluding Observational Study
study (year)
RR (95% CI)
%
Weight
Huang et al. (2015)
0.21 (0.03, 1.58)
1.63
RICCADSA (2016)
0.62 (0.28, 1.38)
10.27
ISAACC (2019)
0.89 (0.67, 1.17)
88.10
Overall, IV (I² = 20.4%, p = 0.284)
0.83 (0.65, 1.08)
100.00
.1
1
10
B. Excluding No. of Participants＜200
study (year)
RR (95% CI)
%
Weight
Cassar et al. (2007)
0.93 (0.78, 1.11)
61.39
Wu et al. (2015)
0.82 (0.53, 1.26)
10.27
RICCADSA (2016)
0.62 (0.28, 1.38)
2.96
ISAACC (2019)
0.89 (0.67, 1.17)
25.37
Overall, IV (I² = 0.0%, p = 0.757)
0.90 (0.78, 1.03)
100.00
.1
1
10
C. Excluding NOS score＜9
study (year)
RR (95% CI)
%
Weight
Milleron et al. (2004)
0.24 (0.09, 0.63)
25.53
RICCADSA (2016)
0.62 (0.28, 1.38)
29.79
ISAACC (2019)
0.89 (0.67, 1.17)
44.68
Overall, DL (I² = 71.0%, p = 0.032)
0.57 (0.28, 1.17)
100.00
.1
1
10
D. Excluding Follow-up Duration≤36 months
study (year)
RR (95% CI)
%
Weight
Milleron et al. (2004)
0.24 (0.09, 0.63)
1.98
Cassar et al. (2007)
0.93 (0.78, 1.11)
59.13
Wu et al. (2015)
0.82 (0.53, 1.26)
9.90
Leão et al. (2016)
0.87 (0.31, 2.46)
1.71
RICCADSA (2016)
0.62 (0.28, 1.38)
2.85
ISAACC (2019)
0.89 (0.67, 1.17)
24.44
Overall, IV (I² = 39.1%, p = 0.145)
0.87 (0.76, 1.00)
100.00
.1
1
10
E. Excluding Lost to Follow-up Rate＞5%
study (year)
RR (95% CI)
%
Weight
Capodanno et al. (2014)
0.18 (0.04, 0.80)
2.06
Huang et al. (2015)
0.21 (0.03, 1.58)
1.12
Wu et al. (2015)
0.82 (0.53, 1.26)
24.64
Leão et al. (2016)
0.87 (0.31, 2.46)
4.24
RICCADSA (2016)
0.62 (0.28, 1.38)
7.09
ISAACC (2019)
0.89 (0.67, 1.17)
60.84
Overall, IV (I² = 22.8%, p = 0.262)
0.81 (0.65, 1.00)
100.00
.1
1
10

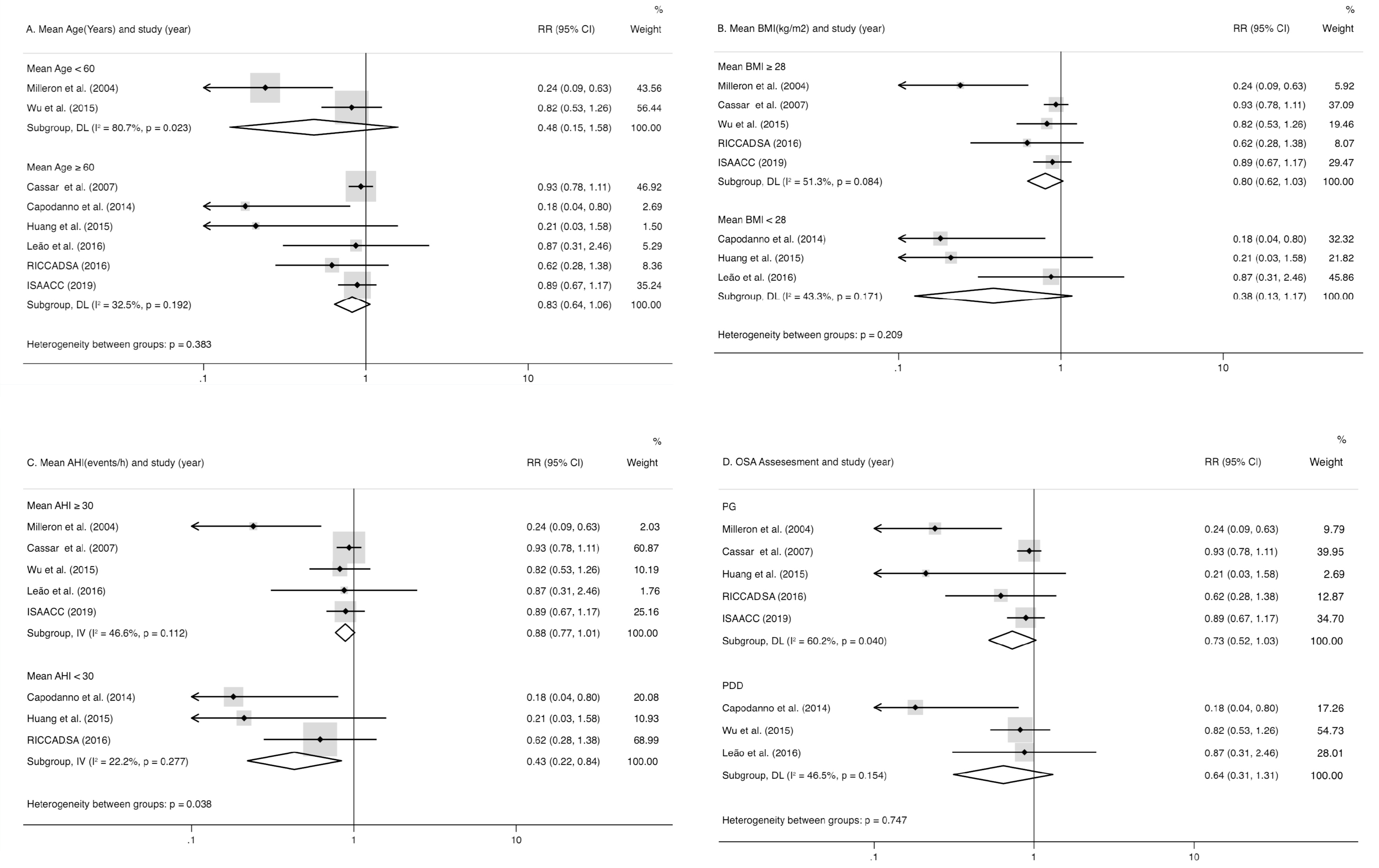

A. Mean Age(Years) and study (year)
RR (95% CI)
%
Weight
Mean Age < 60
Milleron et al. (2004) 0.24 (0.09, 0.63) 43.56
Wu et al. (2015) 0.82 (0.53, 1.26) 56.44
Subgroup, DL (I² = 80.7%, p = 0.023) 0.48 (0.15, 1.58) 100.00
Mean Age ≥ 60
Cassar et al. (2007) 0.93 (0.78, 1.11) 46.92
Capodanno et al. (2014) 0.18 (0.04, 0.80) 2.69
Huang et al. (2015) 0.21 (0.03, 1.58) 1.50
Leão et al. (2016) 0.87 (0.31, 2.46) 5.29
RICCADSA (2016) 0.62 (0.28, 1.38) 8.36
ISAACC (2019) 0.89 (0.67, 1.17) 35.24
Subgroup, DL (I² = 32.5%, p = 0.192) 0.83 (0.64, 1.06) 100.00
Heterogeneity between groups: p = 0.383
.1
1
10
B. Mean BMI(kg/m2) and study (year)
RR (95% CI)
%
Weight
Mean BMI ≥ 28
Milleron et al. (2004) 0.24 (0.09, 0.63) 5.92
Cassar et al. (2007) 0.93 (0.78, 1.11) 37.09
Wu et al. (2015) 0.82 (0.53, 1.26) 19.46
RICCADSA (2016) 0.62 (0.28, 1.38) 8.07
ISAACC (2019) 0.89 (0.67, 1.17) 29.47
Subgroup, DL (I² = 51.3%, p = 0.084) 0.80 (0.62, 1.03) 100.00
Mean BMI < 28
Capodanno et al. (2014) 0.18 (0.04, 0.80) 32.32
Huang et al. (2015) 0.21 (0.03, 1.58) 21.82
Leão et al. (2016) 0.87 (0.31, 2.46) 45.86
Subgroup, DL (I² = 43.3%, p = 0.171) 0.38 (0.13, 1.17) 100.00
Heterogeneity between groups: p = 0.209
.1
1
10
C. Mean AHI(events/h) and study (year)
RR (95% CI)
%
Weight
Mean AHI ≥ 30
Milleron et al. (2004) 0.24 (0.09, 0.63) 2.03
Cassar et al. (2007) 0.93 (0.78, 1.11) 60.87
Wu et al. (2015) 0.82 (0.53, 1.26) 10.19
Leão et al. (2016) 0.87 (0.31, 2.46) 1.76
ISAACC (2019) 0.89 (0.67, 1.17) 25.16
Subgroup, IV (I² = 46.6%, p = 0.112) 0.88 (0.77, 1.01) 100.00
Mean AHI < 30
Capodanno et al. (2014) 0.18 (0.04, 0.80) 20.08
Huang et al. (2015) 0.21 (0.03, 1.58) 10.93
RICCADSA (2016) 0.62 (0.28, 1.38) 68.99
Subgroup, IV (I² = 22.2%, p = 0.277) 0.43 (0.22, 0.84) 100.00
Heterogeneity between groups: p = 0.038
.1
1
10
D. OSA Assesesment and study (year)
RR (95% CI)
%
Weight
PG
Milleron et al. (2004) 0.24 (0.09, 0.63) 9.79
Cassar et al. (2007) 0.93 (0.78, 1.11) 39.95
Huang et al. (2015) 0.21 (0.03, 1.58) 2.69
RICCADSA (2016) 0.62 (0.28, 1.38) 12.87
ISAACC (2019) 0.89 (0.67, 1.17) 34.70
Subgroup, DL (I² = 60.2%, p = 0.040) 0.73 (0.52, 1.03) 100.00
PDD
Capodanno et al. (2014) 0.18 (0.04, 0.80) 17.26
Wu et al. (2015) 0.82 (0.53, 1.26) 54.73
Leão et al. (2016) 0.87 (0.31, 2.46) 28.01
Subgroup, DL (I² = 46.5%, p = 0.154) 0.64 (0.31, 1.31) 100.00
Heterogeneity between groups: p = 0.747
.1
1
10

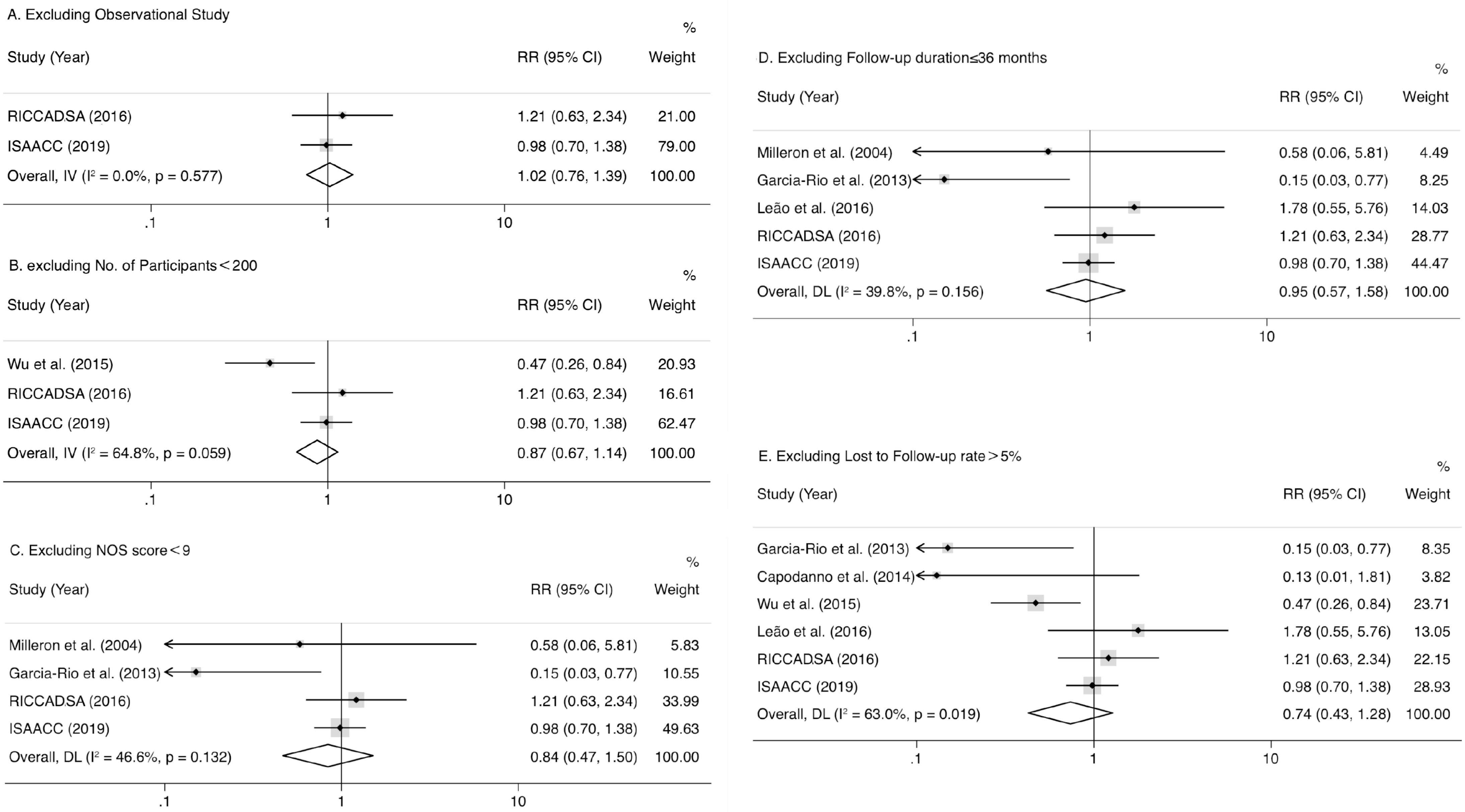

A. Excluding Observational Study
%
Study (Year)
RR (95% CI)
Weight
RICCADSA (2016)
1.21 (0.63, 2.34)
21.00
ISAACC (2019)
0.98 (0.70, 1.38)
79.00
Overall, IV (I² = 0.0%, p = 0.577)
1.02 (0.76, 1.39)
100.00
.1
1
10
B. excluding No. of Participants < 200
%
Study (Year)
RR (95% CI)
Weight
Wu et al. (2015)
0.47 (0.26, 0.84)
20.93
RICCADSA (2016)
1.21 (0.63, 2.34)
16.61
ISAACC (2019)
0.98 (0.70, 1.38)
62.47
Overall, IV (I² = 64.8%, p = 0.059)
0.87 (0.67, 1.14)
100.00
.1
1
10
C. Excluding NOS score < 9
%
Study (Year)
RR (95% CI)
Weight
Milleron et al. (2004)
0.58 (0.06, 5.81)
5.83
Garcia-Rio et al. (2013)
0.15 (0.03, 0.77)
10.55
RICCADSA (2016)
1.21 (0.63, 2.34)
33.99
ISAACC (2019)
0.98 (0.70, 1.38)
49.63
Overall, DL (I² = 46.6%, p = 0.132)
0.84 (0.47, 1.50)
100.00
.1
1
10
D. Excluding Follow-up duration≤36 months
%
Study (Year)
RR (95% CI)
Weight
Milleron et al. (2004)
0.58 (0.06, 5.81)
4.49
Garcia-Rio et al. (2013)
0.15 (0.03, 0.77)
8.25
Leão et al. (2016)
1.78 (0.55, 5.76)
14.03
RICCADSA (2016)
1.21 (0.63, 2.34)
28.77
ISAACC (2019)
0.98 (0.70, 1.38)
44.47
Overall, DL (I² = 39.8%, p = 0.156)
0.95 (0.57, 1.58)
100.00
.1
1
10
E. Excluding Lost to Follow-up rate > 5%
%
Study (Year)
RR (95% CI)
Weight
Garcia-Rio et al. (2013)
0.15 (0.03, 0.77)
8.35
Capodanno et al. (2014)
0.13 (0.01, 1.81)
3.82
Wu et al. (2015)
0.47 (0.26, 0.84)
23.71
Leão et al. (2016)
1.78 (0.55, 5.76)
13.05
RICCADSA (2016)
1.21 (0.63, 2.34)
22.15
ISAACC (2019)
0.98 (0.70, 1.38)
28.93
Overall, DL (I² = 63.0%, p = 0.019)
0.74 (0.43, 1.28)
100.00
.1
1
10

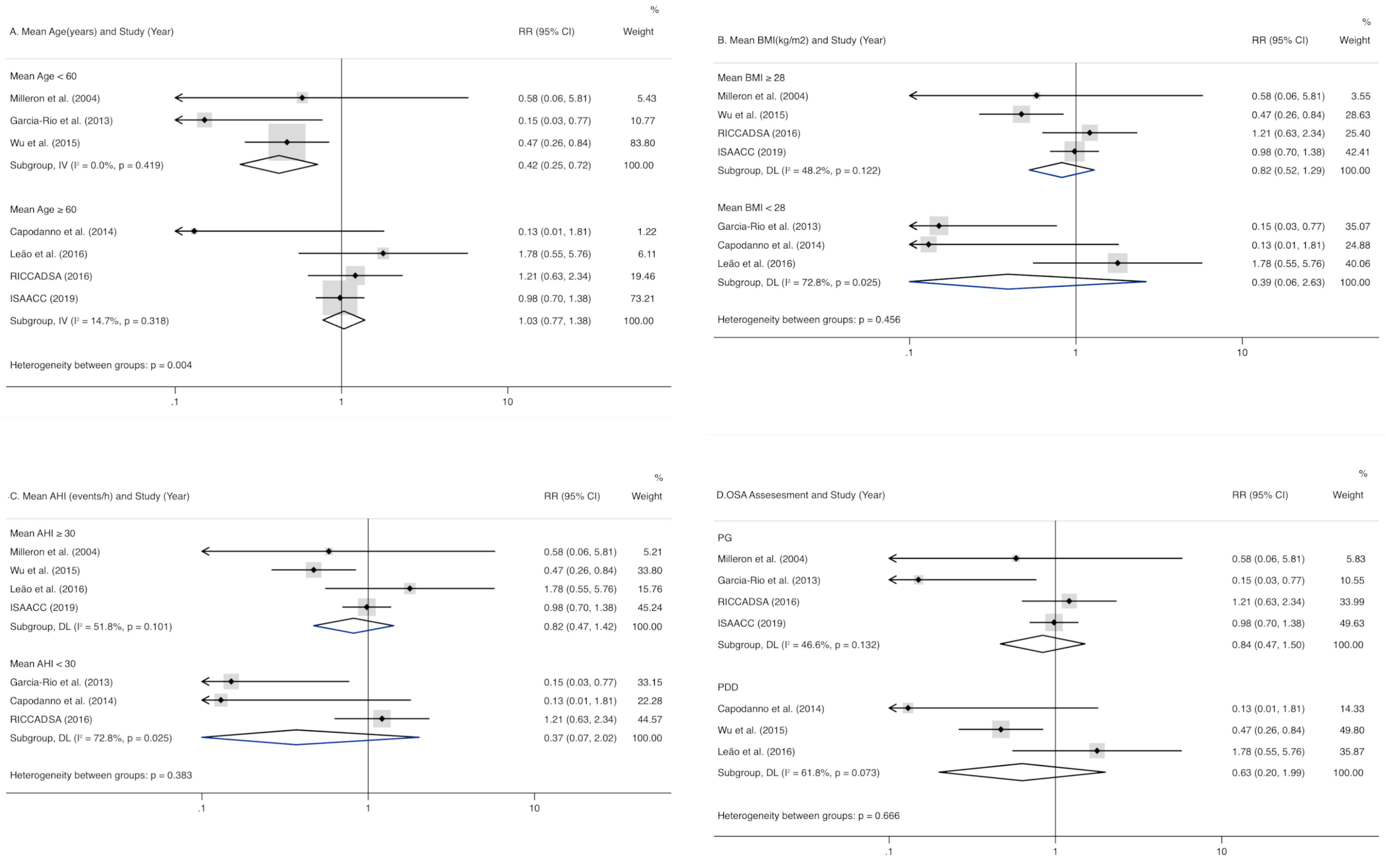
A. Mean Age(years) and Study (Year)
RR (95% CI)
% Weight
Mean Age < 60
Milleron et al. (2004) 0.58 (0.06, 5.81) 5.43
Garcia-Rio et al. (2013) 0.15 (0.03, 0.77) 10.77
Wu et al. (2015) 0.47 (0.26, 0.84) 83.80
Subgroup, IV (I² = 0.0%, p = 0.419) 0.42 (0.25, 0.72) 100.00
Mean Age ≥ 60
Capodanno et al. (2014) 0.13 (0.01, 1.81) 1.22
Leão et al. (2016) 1.78 (0.55, 5.76) 6.11
RICCADSA (2016) 1.21 (0.63, 2.34) 19.46
ISAACC (2019) 0.98 (0.70, 1.38) 73.21
Subgroup, IV (I² = 14.7%, p = 0.318) 1.03 (0.77, 1.38) 100.00
Heterogeneity between groups: p = 0.004
.1
1
10
B. Mean BMI(kg/m2) and Study (Year)
RR (95% CI)
% Weight
Mean BMI ≥ 28
Milleron et al. (2004) 0.58 (0.06, 5.81) 3.55
Wu et al. (2015) 0.47 (0.26, 0.84) 28.63
RICCADSA (2016) 1.21 (0.63, 2.34) 25.40
ISAACC (2019) 0.98 (0.70, 1.38) 42.41
Subgroup, DL (I² = 48.2%, p = 0.122) 0.82 (0.52, 1.29) 100.00
Mean BMI < 28
Garcia-Rio et al. (2013) 0.15 (0.03, 0.77) 35.07
Capodanno et al. (2014) 0.13 (0.01, 1.81) 24.88
Leão et al. (2016) 1.78 (0.55, 5.76) 40.06
Subgroup, DL (I² = 72.8%, p = 0.025) 0.39 (0.06, 2.63) 100.00
Heterogeneity between groups: p = 0.456
.1
1
10
C. Mean AHI (events/h) and Study (Year)
RR (95% CI)
% Weight
Mean AHI ≥ 30
Milleron et al. (2004) 0.58 (0.06, 5.81) 5.21
Wu et al. (2015) 0.47 (0.26, 0.84) 33.80
Leão et al. (2016) 1.78 (0.55, 5.76) 15.76
ISAACC (2019) 0.98 (0.70, 1.38) 45.24
Subgroup, DL (I² = 51.8%, p = 0.101) 0.82 (0.47, 1.42) 100.00
Mean AHI < 30
Garcia-Rio et al. (2013) 0.15 (0.03, 0.77) 33.15
Capodanno et al. (2014) 0.13 (0.01, 1.81) 22.28
RICCADSA (2016) 1.21 (0.63, 2.34) 44.57
Subgroup, DL (I² = 72.8%, p = 0.025) 0.37 (0.07, 2.02) 100.00
Heterogeneity between groups: p = 0.383
.1
1
10
D.OSA Assesesment and Study (Year)
RR (95% CI)
% Weight
PG
Milleron et al. (2004) 0.58 (0.06, 5.81) 5.83
Garcia-Rio et al. (2013) 0.15 (0.03, 0.77) 10.55
RICCADSA (2016) 1.21 (0.63, 2.34) 33.99
ISAACC (2019) 0.98 (0.70, 1.38) 49.63
Subgroup, DL (I² = 46.6%, p = 0.132) 0.84 (0.47, 1.50) 100.00
PDD
Capodanno et al. (2014) 0.13 (0.01, 1.81) 14.33
Wu et al. (2015) 0.47 (0.26, 0.84) 49.80
Leão et al. (2016) 1.78 (0.55, 5.76) 35.87
Subgroup, DL (I² = 61.8%, p = 0.073) 0.63 (0.20, 1.99) 100.00
Heterogeneity between groups: p = 0.666
.1
1
10

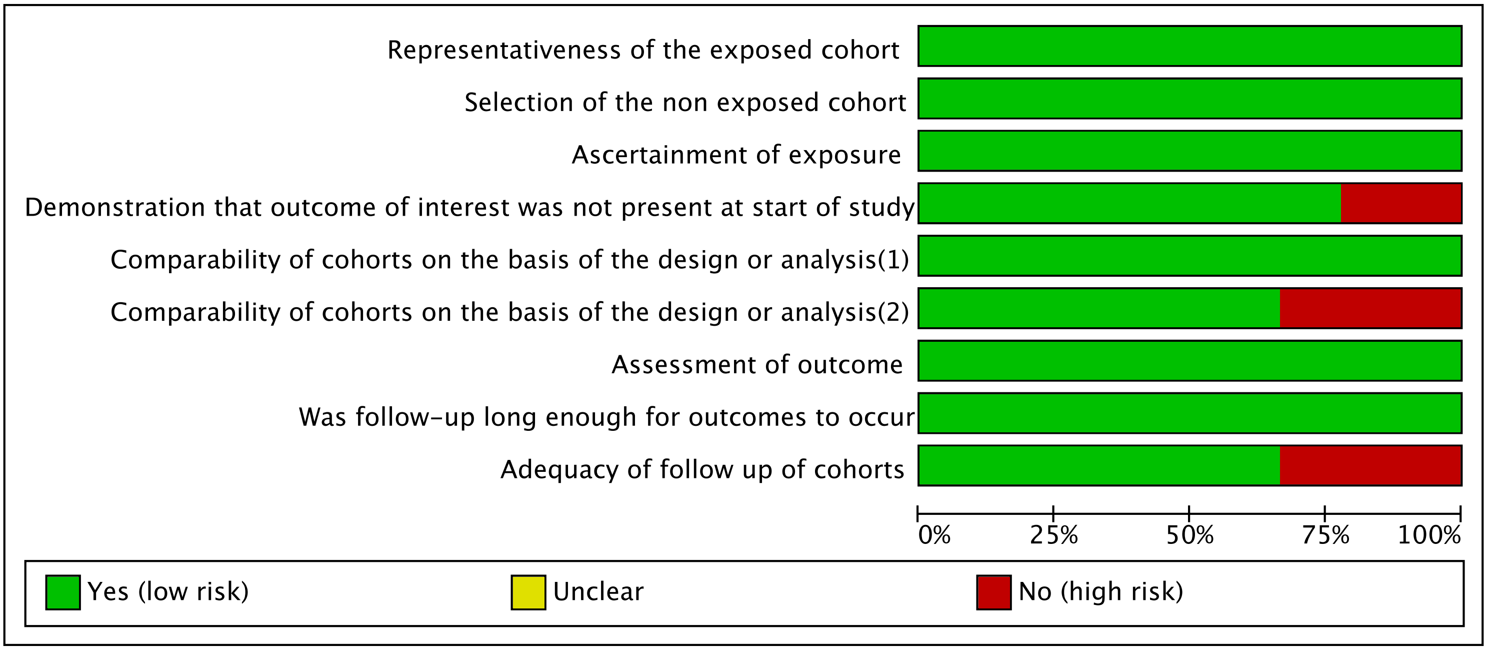
Representativeness of the exposed cohort
Selection of the non exposed cohort
Ascertainment of exposure
Demonstration that outcome of interest was not present at start of study
Comparability of cohorts on the basis of the design or analysis(1)
Comparability of cohorts on the basis of the design or analysis(2)
Assessment of outcome
Was follow-up long enough for outcomes to occur
Adequacy of follow up of cohorts
0%
25%
50%
75%
100%
Yes (low risk)
Unclear
No (high risk)

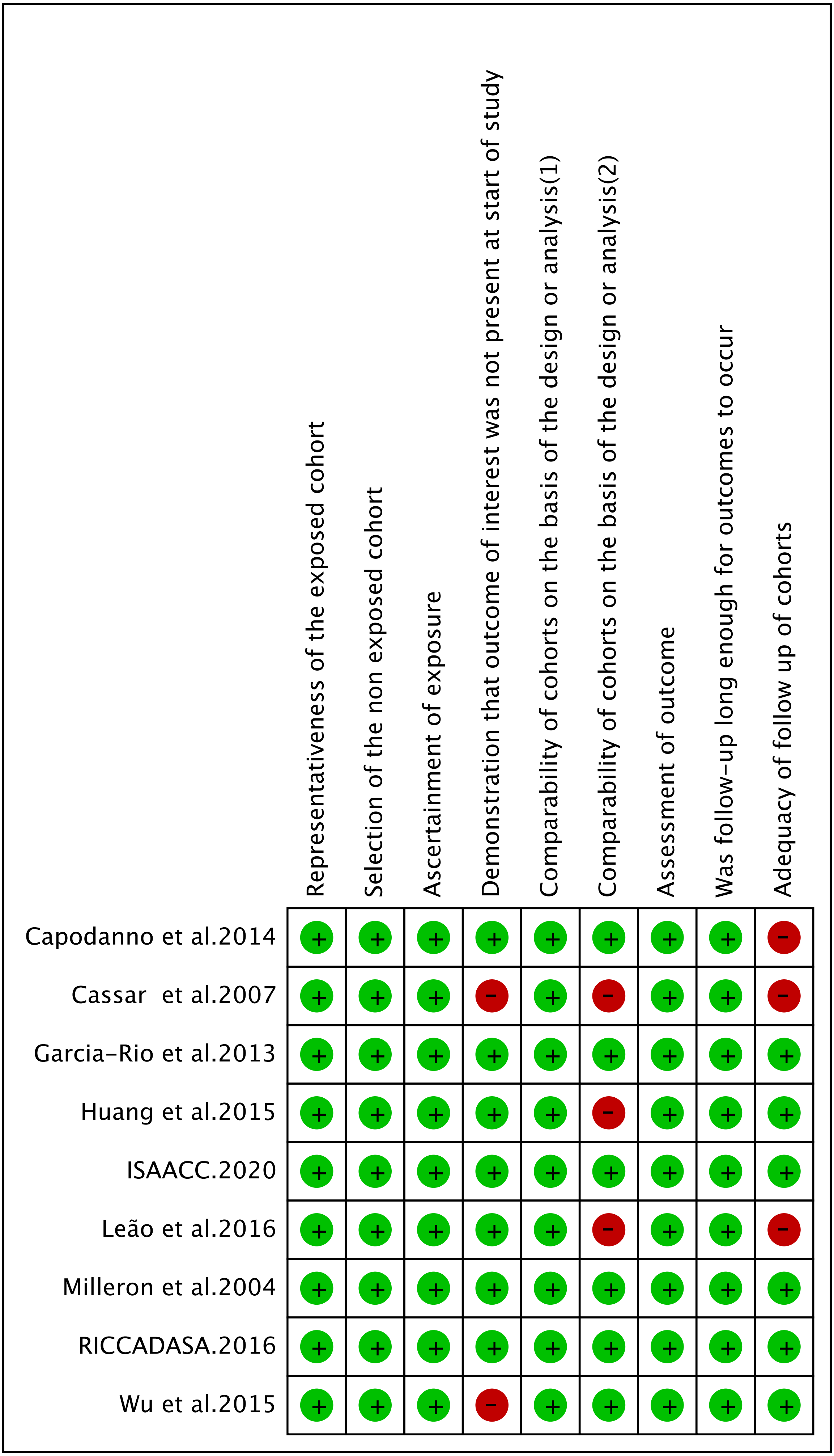
Representativeness of the exposed cohort
Selection of the non exposed cohort
Ascertainment of exposure
Demonstration that outcome of interest was not present at start of study
Comparability of cohorts on the basis of the design or analysis(1)
Comparability of cohorts on the basis of the design or analysis(2)
Assessment of outcome
Was follow-up long enough for outcomes to occur
Adequacy of follow up of cohorts
Capodanno et al.2014
Cassar et al.2007
Garcia-Rio et al.2013
Huang et al.2015
ISAACC.2020
Leão et al.2016
Milleron et al.2004
RICCADASA.2016
Wu et al.2015

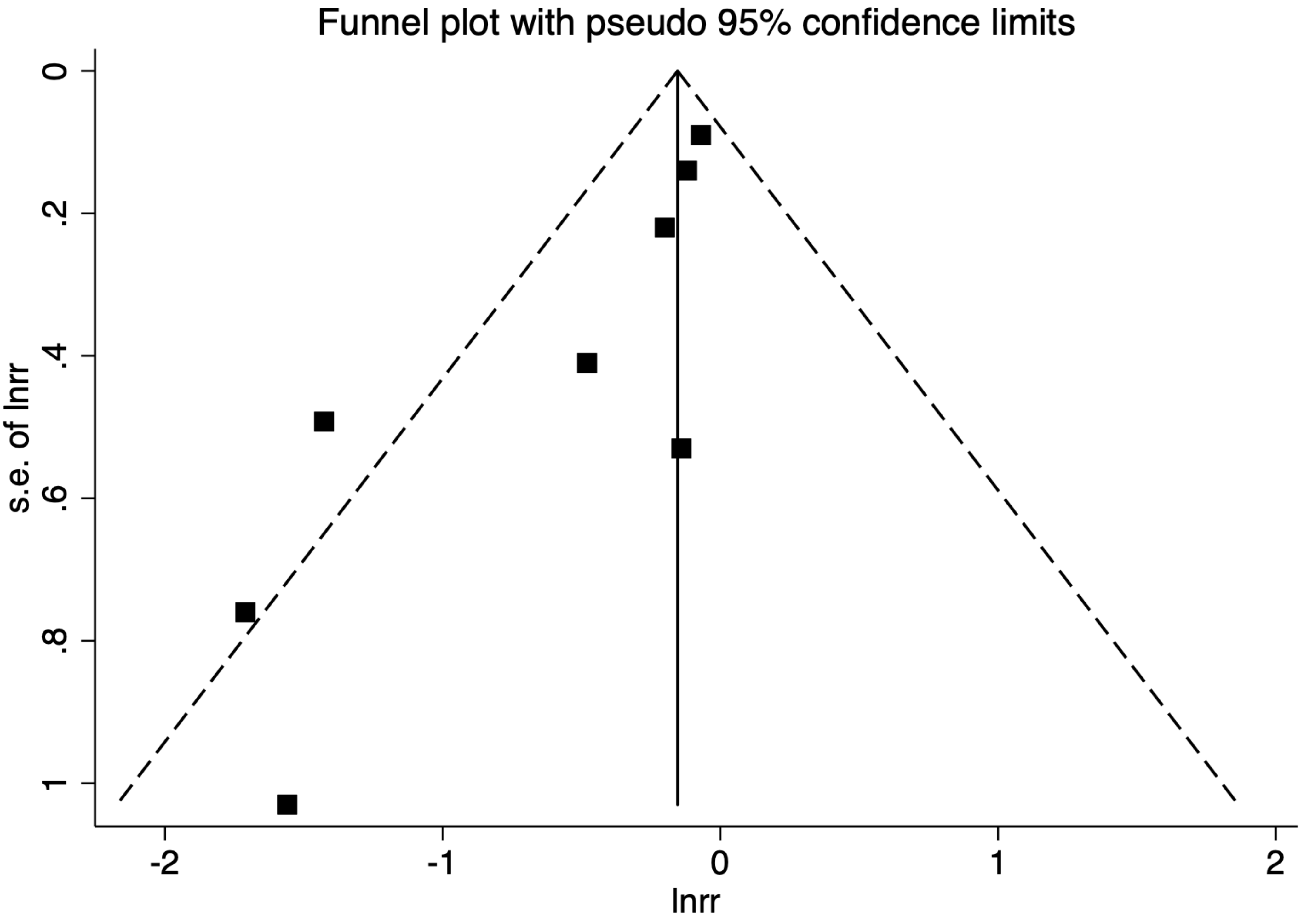
Funnel plot with pseudo 95% confidence limits
s.e. of lnrr
0
.2
.4
.6
.8
1
-2
-1
0
1
2
lnrr

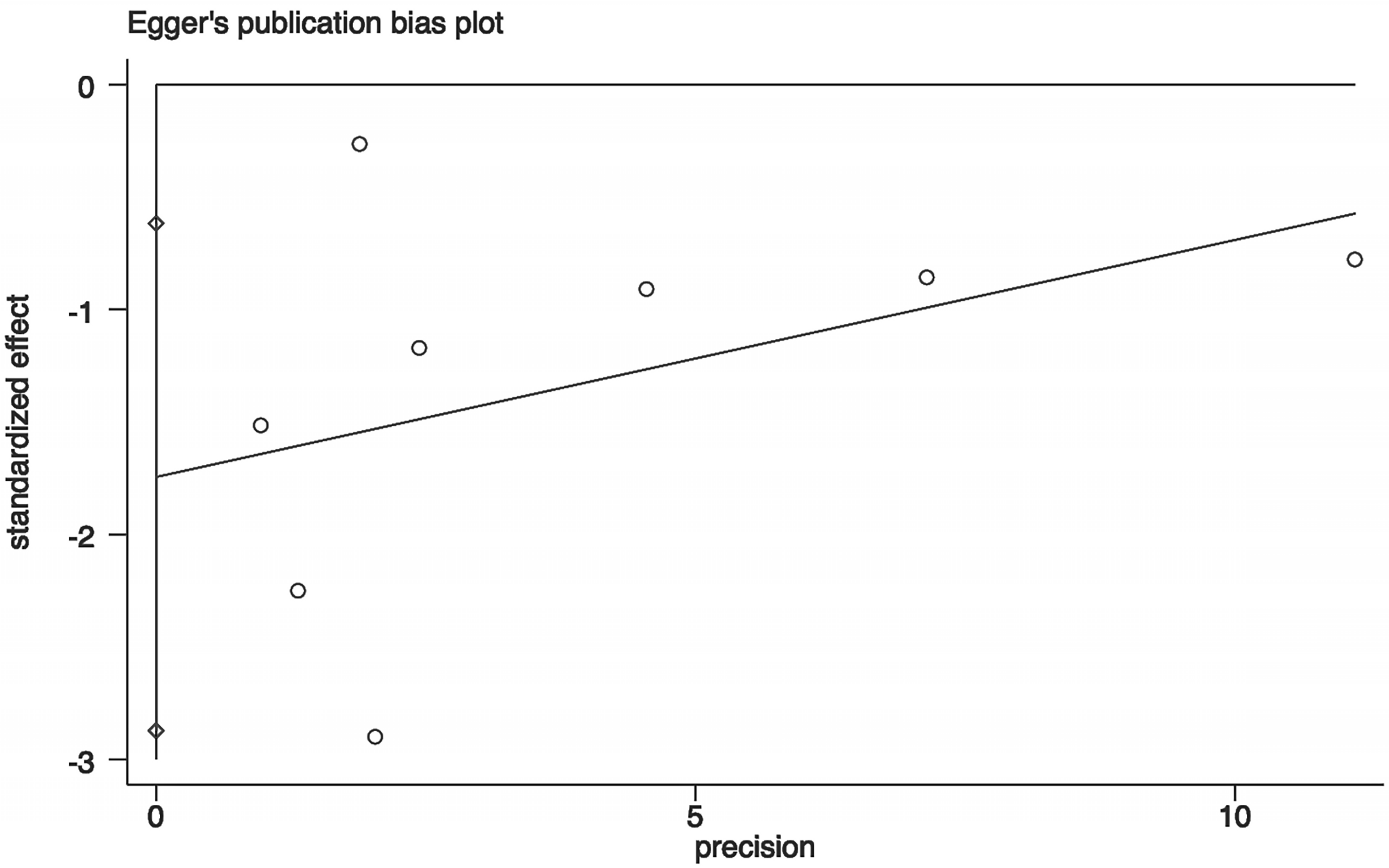
Egger's publication bias plot
standardized effect
0
-1
-2
-3
0
5
10
precision
